# Effect of a Multimodal Pain Therapy Concept Including Intensive Physiotherapy on the Perception of Pain and the Quality of Life of Patients With Chronic Back Pain: A Prospective Observational Multicenter Study Named “RütmuS”

**DOI:** 10.1155/prm/6693678

**Published:** 2025-05-10

**Authors:** Katharina Zaglauer, Andrea Kunsorg, Vanessa Jakob, Lara Görg, Arndt Oehlschlägel, Rainer Riedel, Ursula Marschall, Dieter Welsink, Horst Schuhmacher, Maria Wittmann

**Affiliations:** ^1^Rheinische Hochschule Köln, University of Applied Sciences, Cologne, Germany; ^2^Department of Anaesthesiology and Intensive Care Medicine, University Hospital Bonn, Bonn, Germany; ^3^BARMER Institute for Health Care System Research, Berlin, Germany; ^4^Medicoreha Dr. Welsink Rehabilitation GmbH, Neuss, Germany

**Keywords:** chronic back pain, multimodal pain therapy, physiotherapy, quality of life

## Abstract

**Question and Outcome Measures:** In this study, an intervention group (multimodal therapy for chronic back pain) and a control group (standard outpatient treatment) were compared with regard to the primary endpoint of pain (NRS) at rest and the secondary endpoints pain (NRS) during movement, general health status (Short Form 12 (SF-12)), health-related quality of life (EQ-5D-5L), pain disability index (PDI), Hospital Anxiety and Depression Scale-Germany (HADS-D), and Hannover Functional Ability Questionnaire for Measuring Back Pain-Related Disability (FFbH-R).

**Design and Participants:** The total patient cohort of this prospective observational multicenter study consisted of 477 patients who were initially enrolled in the study from January 2019 to September 2020.

**Intervention:** The intervention group received physiotherapy, pain therapy (pain-therapeutic, body-related, and patient-specific treatment), and control examinations from the responsible physician in a 6-month structured interdisciplinary program. The evaluation points used in the analysis are the baseline survey, 6 months and 12 months after the start of the study.

**Results:** A total of 477 patients (243 in the intervention group and 234 in the control group) were included in the analysis; 42 patients in the intervention group deviated from the eligibility criteria due to insufficient adherence to study participation. Nonetheless, they were included in the analysis in line with the ITT principle. The primary endpoint, pain at rest (NRS), showed greater reductions in the intervention group compared to the control group, with mean differences of −0.492 (95% CI: [−0.866, −0.118], *p* = 0.010) at 6 months (EVA 3) and −0.463 (95% CI: [−0.837, −0.089], *p* = 0.015) at 12 months (EVA 5), respectively. Regarding the secondary endpoints, pain during movement exhibited a significantly greater reduction in the intervention group compared to the control group (*p* < 0.001). Quality of life, measured via the EQ-5D-5L index, improved significantly more in the intervention group than in the control group, as did functional capacity (FFbH-R) and physical health (SF-12 KSK) (*p* < 0.001). In contrast, mental health (SF-12 PSK) declined significantly during the intervention (*p* < 0.001). Disability (PDI) exhibited a significantly greater reduction in the intervention group compared to the control group (*p* < 0.001), whereas anxiety and depression levels (HADS-D) showed only slight changes in both groups, with anxiety being significant at *p* = 0.0164 and depression not significant at *p* = 0.1093. These results underscore the intervention's effectiveness across multiple health dimensions, particularly pain reduction and quality of life.

**Conclusion:** Multimodal pain therapy over a 6-month period is an effective intervention to improve the perception of pain at rest and during movement while enhancing the subjective quality of life. These benefits persist beyond the therapy period, underscoring the intervention's lasting impact.

**Trial Registration:** German Clinical Trials Register: DRKS00015800

## 1. Introduction

Chronic back pain is common in the German population, and affected people are often limited in their daily lives. It is a multifactorial problem characterized by the complex interaction of biological, psychological, and social factors, as well as comorbidities and pain processes [1]. A recent survey in Germany found that 61.3% of respondents reported back pain in the previous 12 months and 15.1% reported chronic back pain in 2020 [2]. In 2022, “an average of 77 days of sick per 100 years of insurance due to back pain” was recorded in Germany [3]; this is the third most common cause of sick leave among employees. A systematic analysis has examined the consequences of low back pain and found that it leads to a reduction in quality of life, sick pay, loss of production, and other medical benefits [4]. In Germany, 14% of the incapacity-for-work days in 2020 were attributed to back pain and the total cost of illness amounted to around 3832 billion euros [5]. In 2021, almost a third of the German population suffered from back pain that required medical treatment [6].

In view of this situation, the effectiveness of therapeutic approaches in the care of patients with chronic back pain is of medical as well as medical–economic importance. Interdisciplinary multimodal pain therapy (MPT) has established itself as an effective therapeutic concept for the treatment of back pain [7–9]. MPT is an interdisciplinary treatment concept that combines various therapeutic approaches and is based on Engel's biopsychosocial pain model [10]. This model assumes that chronic pain has not only physiological, but also psychological and social components that influence its chronification. Psychosocial (yellow flags) and workplace-related factors (blue flags) have a significant influence on the course of the disease [11]. Pain-related cognitions such as fear-avoidance beliefs (e.g., the fear that movement increases pain) can lead to increasing physical inactivity. This reinforces a downward spiral of movement avoidance, loss of function, and increased sensitivity to pain [12]. MPT can effectively interrupt this cycle through targeted patient education, active movement therapy, and relaxation techniques. Studies show that interdisciplinary, activating therapy approaches are more effective than monotherapies that reinforce passive pain behavior [13, 14]. An interdisciplinary approach that integrates medical, pain therapy, and physiotherapy perspectives can help to positively change patients' attitudes toward their illness, increase their quality of life, and sustainably improve their ability to work. A central aim of MPT is to change the patient's perception and attitude toward their illness through targeted patient education and to promote their own activity through self-management. This is achieved, among other things, by consciously promoting body awareness, which is based on the sensorimotor interaction between neuronal processing and motor activity.

Two research groups confirmed the superiority of multimodal therapy compared to “classic conservative therapy” through their comparative studies conducted in the Netherlands [15, 16]. Morfeld et al. compared different multimodal intervention programs for patients with back pain and called for more studies consisting of an intervention and control group to be conducted in order to test which therapy method is superior for patient care in standard treatment settings [17]. Besides the economic aspects, the improvement of the quality of life and reducing the level of pain is essential for the patients.

In view of these positively evaluated interdisciplinary multimodal approaches in the therapy of chronic back issues, BARMER, the second largest statutory health insurance fund in Germany, has concluded a regional selective contract (VMC 0112) on the basis of §140 b SGB V (integrated care) with the aim of interdisciplinary cooperation between orthopedist, a cooperating hospital and a contract rehabilitation facility for the care of chronic nonspecific back pain patients (diagnosis in two consecutive quarters) on a pilot basis, as outpatient multimodal pain treatment is not covered by the catalog of the statutory health insurance funds.

### 1.1. Objectives

The aim of this observational multicenter study was to analyze the effect of a MPT concept including intensive physiotherapy on the perception of pain and the quality of life of patients with chronic back pain. To show the superiority of the multimodal pain treatment concept, this intervention group was compared with a control group from standard care.

## 2. Methods

### 2.1. Design

The study “Back therapy with multimodal pain therapy” (Rückentherapie mit multimodaler Schmerztherapie (RütmuS)) was conducted in a prospective observational and multicenter study design in Germany. Before recruitment began, a positive ethical vote was obtained from the North Rhine Medical Association, Germany. The study was performed in compliance with the Declaration of Helsinki. Furthermore, the RütmuS study was registered with the German Registry for Clinical Studies. Participants were included after written informed consent from January 2019 to September 2020.

### 2.2. Participants

Of the 477 patients enrolled in the study, 243 were included in the intervention group (IV) and 234 in the control group (C). By utilizing multiple imputation to address missing data, we ensured the inclusion of all eligible patients in the analysis, reflecting the full cohorts for both groups. In the intervention group, 42 patients did not meet the adherence requirements; nevertheless, they were retained in the analysis consistent with the ITT approach.

The intervention group comprised patients who participated in an integrated care program for the treatment of chronic back pain, meeting the required therapy session criteria for inclusion. This selective contract to be evaluated regulates integrated care on the basis of §140 b of the German Social Code, Book V (SGB V) with the aim of integrated cooperation between the general practitioners, the cooperation hospital, and the contract rehabilitation facility in the care of patients with chronic back pain. The integrated care contract existed before, at, and after the time of the study between the BARMER health insurance fund and an outpatient rehabilitation network (medNetNeuss). Patients who lived in Neuss (large city in North Rhine-Westphalia) and the surrounding area were assigned to the complex treatment of the existing integrated care contract by the general practitioners or specialist. Only after the patients had agreed to participate in the integrated care program were they asked to take part in this study. The control group of the RütmuS study consisted of BARMER insurance members who received standard care. The general practitioners and specialists were located in the Cologne/Bonn area, a distinct region within North Rhine-Westphalia, and the patients therefore had no access to treatment via the integrated care contract. After the general practitioners or specialists had checked the inclusion and exclusion criteria for the study, the patients were asked whether they would like to take part in the study as control group participants (effort of the interviews by the study team). There was no further standardized contact with the physician after successful inclusion in the control group.

The same inclusion and exclusion criteria were applied to both study groups. Adults aged between 18 and 85 years who were insured with BARMER health insurance were included. In addition, at least one of the following five ICD diagnoses had to be present: M42, osteochondrosis of the spine; M50, cervical disc damage; M51, other disc damage; M53, other diseases of the spine and back, not elsewhere classified; and M54, back pain. Furthermore, at least one of these designated ICD codes had to be coded by the treating physician in two consecutive quarters to have the characteristics of chronic back pain. Informed consent was also required before data collection.

Without providing consent, the patient could not be enrolled in the study. The following exclusion criteria were specified as follows: Patients were excluded if they had insufficient German language skills, a tumor disease, contraindications for physiotherapeutic interventions (e.g., traumatic paraplegia, risk of circulatory failure, and recent fractures), or an existing need for long-term care. The existing need for care was defined in the exclusion criteria as follows: “an existing degree of care.” Social Code Book XI (SGB XI) states that people with statutory insurance are entitled to a degree of care following an assessment by the medical service. The care level determines the severity of the impairment. People with such a categorization were excluded from the study, as it can be assumed that they cannot adequately participate in the integrated care concept due to their limitations. Furthermore, patients with a current application for a disability pension were also excluded due to the missing comparability. If the potential study participants had taken part in a rehabilitation program for chronic back pain in the previous year, they were also excluded due to lack of comparability.

### 2.3. Intervention

The patients in the intervention group participated in the integrated care contract for chronic back pain. The treatment team of medical and nonmedical therapists worked together in partnership and thus enabled the multimodal therapy approach. In the outpatient setting, the orthopedist or general practitioner, referred to here as the patient-guiding physician, was responsible for enrolling patients in the integrated care contract, conducting diagnostics, and performing specified checks (control examination) for their enrolled patients. In addition, the patient-guiding physician carried out a follow-up and final examination. The integrated care treatment primarily focused on pain therapy, movement-related care, and patient-centered approaches. Compared to standard care, the multimodal therapy program offered the insured clear, coordinated, and guideline-based care pathways. To optimally address the treatment needs of the patients, a weekly case conference was held among all participating service providers. Here, the cases were discussed between physicians and physiotherapists, and the treatment management was documented in a standardized way.

The multimodal pain concept consists of a pain analysis to exclude specific back pain, documentation of biopsychosocial chronification factors, of a physiotherapeutic treatment (complex outpatient therapy (COT)) adapted according to the patient's needs and a follow-up examination. The aim was to strengthen self-control through mutual exchange. This exchange took place in the standardized therapy meetings between the interdisciplinary team of therapists and the patients. The primary topics focused on self-responsible health behavior and maintaining control in the face of psychosocial stress.

Table 1 shows the timeline and the different steps of the care pathway with the integrated care contract. With a total of 44 therapeutic units involving direct patient contact, physiotherapy formed the largest therapeutic part of the MPT. In Phase II (COT), the following interventions were implemented across 20 therapeutic units (each lasting 120 min) over a period of 10 weeks: training in small groups; active physiotherapy in individual sessions and physical therapy measures; along with individual, patient-centered physiotherapeutic rehabilitation exercises. The 120 min was predominantly allocated to 30 min of activating physiotherapy and 90 min of training therapy.

The 30-min activating physiotherapy was carried out exclusively in an individual setting (1:1), i.e., one therapist worked with one patient at a time. The individual target agreements and the therapy plan were developed on the basis of the physiotherapeutic diagnosis, taking into account the patient's subjective understanding of the illness and perception of stress. The therapy was based on the patient's physical performance requirements and was gradually adapted during Phase II using exercise parameters. In the initial phase of therapy, the patient was taught perception-oriented, back-relieving exercises to improve body awareness and relief strategies. Passive therapy methods (e.g., manual therapy or physical measures) were used to supplement active therapy, depending on the indication.

The 90-min training therapy was a movement-based therapy session to restore functional stability, which was carried out on the training area using therapeutic training equipment. The patients trained simultaneously according to individual, guideline-based training plans. This physiotherapeutic training therapy is based on the principles of activation according to the current National Care Guideline for Nonspecific Low Back Pain [11], which recommends early physical activity to promote functional improvements.

Each patient received 15 min of targeted 1:1 supervision by a physiotherapist, during which individual corrections, load control guidelines, and exercise adaptations were made. In the remaining 75 min, the patients trained independently while the physiotherapist supervised the entire training area and provided support where necessary.

In addition to these therapy units, the patients took part in three separate 30-min physiotherapy training sessions. These took place in groups with a maximum of eight participants and conveyed theoretical and educational content based on the biopsychosocial model of pain therapy. The aim was to promote self-efficacy through patient education, movement promotion, and pain management strategies, as well as to change the perception of illness by activating and improving body awareness.

The sessions imparted knowledge about the biomechanical function of the spine, the causes, and influencing factors of chronic back pain and evidence-based strategies for self-help. A central component was the exchange of experiences between patients and therapists to collaboratively develop effective coping strategies.

In Phase III, the individually tailored physiotherapeutic rehabilitation training was continued for 24 therapeutic units. This phase aimed to stabilize the patient and, if necessary, to increase the level of exercise according to the demands of daily life or work.

In addition to physiotherapeutic treatment, the patient also received balanced pain therapy during the intervention. The pain therapy carried out by medical pain therapists in the intervention group included the following services: 1× basic pain assessment, 1× follow-up pain assessment, 8× group sessions “Pain in conversation.” The pain therapist analyzed the causes of pain to rule out specific back pain and identify psychosocial factors that promote the chronification of pain. On this basis, the COT and aftercare were individually adapted. The identification of psychosocial risk factors was based on established models of pain management and was divided into two main categories: psychological factors and social factors. The psychosocial factors to be identified included fear-avoidance beliefs, catastrophizing, depression, and helplessness, as well as pain-related beliefs and attitudes [12, 18]. The potential social factors that were assessed by the pain therapist included social isolation and lack of support, secondary disease gain, workplace-related stress, and social modeling, among others.

After this assessment, the patient was trained by the medical pain therapist based on the following content:

• Theoretical basis: the biopsychosocial pain model according to Engel formed the basis of the treatment, with the aim of strengthening the patient's self-control and self-efficacy through educational, cognitive, and physical interventions [10].
• Knowledge transfer about back pain, disease management, and body-related exercises. For example, progressive muscle relaxation according to Jacobson [19], which has proven to be effective in reducing pain intensity and muscle tension [20].
• Promotion of self-responsible health behavior in dealing with the body, taking into account individual psychosocial stress.
• Further therapeutic modules: patient education, behavioral therapy interventions, stress management, and physical activation to reduce psychosocial risk factors.

### 2.4. Control Group Comparison to Intervention Treatment

The control group received the usual standard of care in the care region, which was highly variable. Treatment was carried out according to medical indication, in particular by general practitioners and orthopedists, and could include the following measures:

• Drug therapy
• Invasive therapy, e.g., infiltrations
• Referral for surgical procedures
• Physical therapy, e.g., massages, heat, and electrotherapy
• Individual physiotherapy treatments without a standardized program
• Prescription of other remedies (e.g., occupational therapy) or AIDS
• And other procedures

In the control group, pain therapy was provided exclusively as part of the usual medical care. This primarily included pharmacological interventions (e.g., analgesics, antidepressants) and, depending on the medical indication, invasive procedures (e.g., injections) or referral for surgery. Physiotherapeutic individual treatments and physical therapies (e.g., heat and electrotherapy) were carried out according to a doctor's prescription, without a standardized framework for prescribing remedies.

In contrast to the intervention group, there was no systematic recording of psychosocial risk factors and no interdisciplinary case discussion. Furthermore, educational group interventions for pain management were not part of standard care.

The present study was designed as an observational cohort study to reflect real care structures without interfering with routine care. Therefore, measurements were taken independently of the treating professionals to avoid bias.  Table 2 schematically lists the various treatment components of the intervention and control group.

### 2.5. Outcome Measures

#### 2.5.1. Primary Outcome

The primary endpoint of the RütmuS study was the change in patients' subjective perception of pain at rest, measured over the course of the study using the Numeric Rating Scale (NRS), which ranges from 0 (no pain) to 10 (worst pain imaginable). Given the characteristics of the NRS, it was treated as a ratio-scaled variable for analysis. The focus on the change in pain at rest reflects the dynamic nature of therapeutic progress and provides a robust measure of treatment efficacy. Pain at rest was selected as a primary focus because the NRS is a validated and widely accepted tool, enabling standardized assessment and comparison across studies. Furthermore, reductions in pain at rest are critical for improving patients' quality of life and psychological well-being, as chronic pain often leads to emotional distress and functional impairments. MPT, as applied in this study, frequently prioritizes alleviating pain at rest to facilitate self-directed health behaviors and effective coping strategies.

#### 2.5.2. Secondary Outcome

In addition to pain at rest, the study also assessed secondary outcomes, including pain during movement, quality of life (SF-12 and EQ-5D-5L), functional limitations (FFbH-R), pain-related disability (pain disability index [PDI]), and psychological factors such as anxiety and depression (HADS-D). These outcomes provide a broader understanding of the intervention's impact.

The EQ-5D-5L questionnaire [21] assesses five dimensions—mobility, self-care, usual activities, pain/discomfort, and anxiety/depression—each rated on five severity levels. Patient responses generate a health state that is summarized as an index value, reflecting health-related quality-of-life preferences in the general population. This index ranges from 0 (0%) to 1 (100%), with possible values below 0 indicating states worse than death.

The Short Form 12 (SF-12) [22] questionnaire assesses health-related quality of life through 12 self-reported items with varying response options. It generates two summary scales: The Physical Health Summary Scale (KSK) and the Mental Health Summary Scale (PSK), each based on six items. Scores are calculated using regression coefficients, with higher scores indicating better health status.

The PDI [23] measures self-reported pain-related disability in daily life using seven items, each rated on a scale from 0 to 10. A simple sum score is calculated, with lower scores indicating less disability. The maximum possible score is 70, representing the highest level of pain-related impairment.

The Hospital Anxiety and Depression Scale-Germany (HADS-D) [24] is a validated self-assessment tool designed to measure anxiety, depression, and psychological distress, particularly in patients with physical illnesses. The scale comprises 14 items, split into two subscales—anxiety and depression—each containing seven items with four response options. Scores for each subscale are summed, with higher values indicating greater symptom severity. Scores of 11 or above are considered indicative of clinically significant symptoms, scores of 7 or below as nonsignificant, and scores between 8 and 10 as borderline. The maximum possible score for each subscale is 21.

The Hannover Functional Ability Questionnaire for Measuring Back Pain-Related Disability (FFbH-R) [25] is a self-assessment tool designed to evaluate functional limitations in daily life specific to patients with back pain. The questionnaire consists of 12 items, each with three response options scored from 0 to 2. The total score is calculated by summing these values and is subsequently converted into a functional capacity percentage ranging from 0% to 100%, where 0% represents maximum functional impairment and 100% indicates full functional capacity.

For enhanced comparability between the intervention and control groups, we collected baseline data encompassing demographic characteristics and clinical variables. Demographic characteristics included age (years), gender (*n* (%) female/*n* (%) male), body mass index (BMI, kg), education level (*n* (%) with postsecondary, secondary, or primary education), and marital status (*n* (%) married or cohabiting, single, widowed, or divorced/separated). Clinical characteristics comprised pain at rest at baseline (NRS), pain during movement at baseline (NRS), fingertip-to-floor (FTF) distance at baseline (cm), sport and physical activity (minutes/week), quality of life at baseline (EQ-5D-5L Index), and back pain diagnoses according to ICD-10 codes (*n* (%) for M42, M50, M51, M53, M54). These variables are summarized in Table 3.

#### 2.5.3. Study Timeline and Data Collection Overview

The data collection of the patients was carried out by the independent Institute for Medical Economics & Medical Care Research of the Rheinische Hochschule Köln, University of Applied Sciences, Cologne. The intervention and patient treatment were conducted independently of the RütmuS study. Patients treated according to the integrated care contract were assigned to treatments by their treating physicians. The physicians only asked the patients (of both groups) if they would like to participate in the observatory trial and filled out a short medical questionnaire. The questionnaire and the informed consent were transferred to the Rheinische Hochschule. The outcome assessments were carried out by the trial staff at the Rheinische Hochschule, who were not involved in the medical treatment of the patients.

The evaluation dates were set as follows:

• EVA 1: Baseline (first week)
• EVA 2: 12 weeks (3 months) after inclusion (phone survey)
• EVA 3: 26 weeks (6 months) after inclusion
• EVA 4: 39 weeks (9 months) after inclusion (phone survey)
• EVA 5: 52 weeks (12 months) after inclusion.

Data on NRS at rest, NRS during movement, and EQ-5D-5L were systematically collected across all five evaluation time points (see Table 4). By design, the assessments at EVA 1, 3, and 5 were comprehensive, relying on written questionnaires, and intentionally differed substantially from those at EVA 2 and EVA 4, which were conducted via telephone. These telephone check-ins at EVA 2 and EVA 4 were deliberately kept rudimentary, using interviewer-led questioning and lacking the depth of the written assessments, making them unsuitable for direct comparison with the primary data collection points. Accordingly, they were excluded from the analysis and instead served as planned interim check-ins to sustain patient engagement and provide support throughout the study.

### 2.6. Sample Size Calculation

The calculation of the required number of patients for each study group was based on the expected effect sizes. Cohen's *d* was used as a measure of the effect size, since it is based on the standardized difference between the expected values of the intervention and control groups regarding the primary endpoint. For the determination of the study population, a sample size calculation was carried out on the basis of the difference between the EVA 5 and EVA 1 pain score (according to the NRS at rest, respectively) as the primary endpoint. Taking these data into account and assuming a difference in the reduction in pain perception of medium magnitude (effect *d* = 0.4) between intervention and control groups from EVA 1 to EVA 5, the minimum number of study participants was quantified. To determine the above study population, a significance level of 5% and a power of 80% were specified. The study population was thereby estimated taking into account both one-sided and two-sided test designs for the primary endpoint.

Initially, it was planned to perform the analysis of the primary endpoint based on two subgroups (insured members/insured dependents and insured retirees). The sample size calculation therefore led to the following study population of a minimum total of 480 patients.

### 2.7. Statistical Methods

Statistical analyses were conducted using R Version 4.4.3. Patients were classified into two groups based on their participation in the integrated care contract for chronic back pain therapy: the intervention group (IV) and the standard care group (control, C), as previously described.

Demographic and baseline clinical characteristics were summarized for the entire study population and stratified by group. Continuous variables were reported as mean ± standard deviation (SD), while categorical variables were expressed as absolute and relative frequencies (percentages).

Multiple imputation was employed to address missing values in key outcome variables across the three evaluation time points (EVA 1, 3, and 5). Predictive mean matching was applied to generate multiple complete datasets, leveraging correlations across variables and time points.

The primary analysis focused on changes in NRS scores at rest, while secondary analyses examined NRS scores during movement and other secondary outcomes, including EQ-5D-5L index values, SF-12, PDI, HADS-D, and FFbH-R, from baseline (EVA 1) to the final evaluation (EVA 5).

To evaluate the intervention's impact on pain reduction, an initial mixed-effects model, excluding potential confounders, was constructed to establish a baseline understanding of the intervention's effect [26, 27]. A subsequent extended model incorporated potential confounders, including age, gender, BMI, pain during movement, educational level, marital status, and ICD diagnoses. This comprehensive model provided a robust evaluation of group differences in pain reduction over time. A significance threshold of *p* < 0.05 was applied.

Post hoc pairwise comparisons, adjusted using the Bonferroni method, were performed to identify group differences at each time point. Results were presented as estimated marginal means with 95% confidence intervals and *p* values to quantify the statistical significance of group differences.

To visualize the data, box-and-whisker plots were generated for NRS scores at rest and during movement, as well as for other secondary outcomes, including EQ-5D-5L index values, SF-12, PDI, HADS-D, and FFbH-R, across the three evaluation time points (EVA 1, 3, 5). These plots depicted distributions using the five-number summary (minimum, maximum, median, and quartiles 1 and 3). Complementary summary statistics, including mean, SD, minimum, and maximum, were also provided.

In addition, Spearman's rank correlation tests were applied to explore the relationship between the changes in NRS score at rest/during movement and the change in EQ-5D-5L index value. Correlations of 0.1, 0.3, and 0.5 were used as thresholds for weak, moderate, and strong correlations, respectively [28].

## 3. Results

### 3.1. Flow of Participants

**Figure 1:**
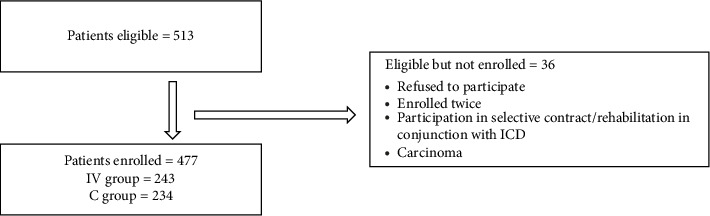
Flowchart illustrating the patients eligible and patients enrolled.

Of the 513 eligible patients, 477 were enrolled, with 243 assigned to the intervention (IV) group and 234 to the control (C) group (Figure 1).

### 3.2. Compliance With Trial Method

Forty-two patients in the intervention group did not complete the intervention as required but were retained in the analysis in accordance with the ITT principle.

### 3.3. Characteristics of Participants

The two groups were mostly similar regarding their baseline characteristics at admission to the study (Table 3). Exceptions are pain during movement, which was significantly higher at the start for intervention patients than for control patients. Furthermore, percentages in ICD-10 codes M42 and M51 differed significantly between intervention and control groups and significant differences in education and marital status were observed (Table 3). To account for these baseline differences, potential confounders were incorporated into the analysis, ensuring a more accurate estimation of the intervention's effects.

### 3.4. Effects of the Intervention

#### 3.4.1. Primary Outcome

To evaluate the intervention's impact on pain reduction, two different mixed-effects models were employed, using restricted maximum likelihood (REML) estimation to account for random intercepts at the participant level.

Initially, a mixed-effects model was fitted without considering potential confounders to assess the effect of the intervention on pain reduction. Pain at rest (NRS) was modeled as the dependent variable, with group (intervention vs. control), time (baseline [EVA 1], follow-ups [EVA 3, EVA 5]), and their interaction as fixed effects. A random intercept for participants accounted for repeated measures within individuals.

The analysis revealed significant main effects for time, with reductions in pain scores observed at both EVA 3 (Estimate = −1.928, 95% CI: [−2.240, −1.616], *p* < 0.001) and EVA 5 (Estimate = −1.865, 95% CI: [−2.177, −1.553], *p* < 0.001) compared to baseline (EVA 1). Additionally, significant interaction effects between group and time were found at EVA 3 (Estimate = 1.353, 95% CI: [0.908, 1.780], *p* < 0.001) and EVA 5 (Estimate = 1.294, 95% CI: [0.849, 1.739], *p* < 0.001), indicating that pain reductions differed significantly between the intervention and control groups over time. These findings are presented in Table 5.

Baseline comparisons (EVA 1) showed no significant differences in pain scores between the groups (mean difference = 0.322, 95% CI: [−0.087, 0.751], *p*=0.121). However, at EVA 3, the intervention group reported significantly lower pain scores compared to the control group (mean difference = −1.022, 95% CI: [−1.441, −0.603], *p* < 0.001). This difference was maintained at EVA 5 (mean difference = −0.962, 95% CI: [−1.381, −0.543], *p* < 0.001). These post hoc pairwise comparisons are summarized in Table 6.

The estimated marginal means (Table 7) further illustrate these findings, showing the adjusted mean pain scores for each group at different time points. At EVA 3 and EVA 5, the intervention group consistently demonstrated lower pain scores compared to the control group, highlighting the effectiveness of the intervention in reducing pain over time.

Building upon the findings from the initial mixed-effects model, which excluded potential confounders to provide a baseline understanding of the intervention's effect, a more comprehensive analysis was conducted. This extended model accounted for potential confounders such as age, gender, BMI, pain during movement, educational level, marital status, and ICD diagnoses. Pain scores at rest (NRS) served as the dependent variable, with group (intervention vs. control), time (baseline [EVA 1] and follow-ups [EVA 3, EVA 5]), their interaction, and potential confounders modeled as fixed effects. A random intercept for participants accounted for repeated measures within individuals.

Key findings from the extended model are as follows:

1. Time and Interaction Effects: Pain scores at rest decreased significantly over time in both groups, as indicated by reductions at EVA 3 (estimated effect = −0.818, 95% CI: [−1.130, −0.506], *p* < 0.001) and EVA 5 (estimated effect = −0.769, 95% CI: [−1.081, −0.457], *p* < 0.001) compared to baseline (EVA 1). While this main effect of time was observed across both groups, the trends in interaction effects between group assignment and evaluation time suggest that the intervention group experienced a more pronounced reduction in pain over time compared to the control group. Specifically, at EVA 3, the interaction effect was estimated at 0.440 (95% CI: [−0.021, 0.860], *p*=0.040), and at EVA 5, the estimated effect was 0.411 (95% CI: [−0.065, 0.828], *p*=0.055) (Table 8). These results indicate a potential trend toward greater improvements in the intervention group, although the interaction effect at EVA 5 narrowly missed statistical significance.
2. Role of Confounders: Among the potential confounders, pain scores during movement (NRS during movement) demonstrated a strong association with pain scores at rest (estimated effect = 0.441, 95% CI: [0.396, 0.486], *p* < 0.001) underlining the interrelation of these pain dimensions. Other potential confounders, such as age, gender, BMI, educational level, marital status, and ICD diagnoses, did not show significant associations (*p* > 0.05). Global *p* values are reported for the categorical predictors of educational level and marital status, both of which have more than two levels (Table 8).  Table 9 presents the random effects of the mixed-effects model without confounders, showing variance and SD for participant intercepts and residuals. Including confounders (Table 10) reduces both participant and residual variability, indicating a better model fit by accounting for systematic differences.
3. Post Hoc Analyses: Post hoc comparisons further supported the trends observed in the main effects (see Table 11). At baseline (EVA 1), no significant difference in pain scores at rest was observed between the intervention and control groups (mean difference: −0.053, 95% CI: [−0.435, 0.329], *p*=0.786). At EVA 3, the intervention group exhibited significantly lower pain scores compared to the control group (mean difference: −0.492, 95% CI: [−0.866, −0.117], *p*=0.010). At EVA 5, the intervention group maintained significantly lower pain scores compared to the control group (mean difference: −0.463, 95% CI: [−0.837, −0.089], *p*=0.015).

These findings emphasize the significant reductions in pain scores achieved by the intervention group, particularly at EVA 3 and EVA 5. The results, adjusted for a wide range of confounders, provide robust evidence of the intervention's effectiveness in reducing pain at rest. The 95% confidence intervals underscore the precision of the estimates.

The estimated marginal means (Table 12) offer further insights into the adjusted pain scores at rest for each group across the three evaluation time points (EVA 1, EVA 3, and EVA 5). These means account for the effects of confounding variables, providing a clearer representation of the intervention's impact.

At baseline (EVA 1), the intervention group and control group exhibited similar pain scores at rest, with marginal means of 4.06 (95% CI: [3.474, 4.646]) and 4.11 (95% CI: [3.522, 4.698]), respectively. This confirms the absence of significant baseline differences between the groups. By EVA 3, the intervention group demonstrated a lower marginal mean pain score of 3.24 (95% CI: [2.646, 3.834]) compared to the control group's 3.73 (95% CI: [3.148, 4.312]), reflecting a notable reduction. A similar pattern was observed at EVA 5, with the intervention group maintaining a lower mean score of 3.29 (95% CI: [2.698, 3.882]) relative to the control group's 3.75 (95% CI: [3.166, 4.33]).

**Figure 2:**
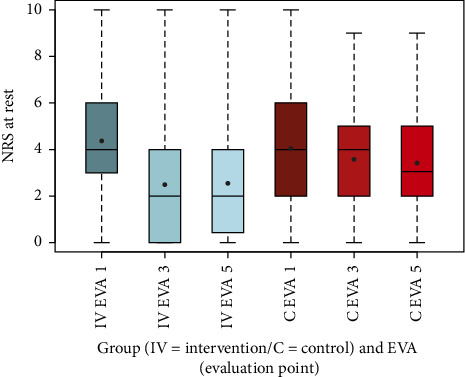
Comparison of NRS at rest intervention (IV) group vs. control (C) group.

To visually represent the above findings, Figure 2 displays box-and-whisker plots for NRS scores at rest across the intervention and control groups at EVA 1, EVA 3, and EVA 5. These plots illustrate the distribution of pain scores, providing context for the group differences observed in the model.

#### 3.4.2. Secondary Outcomes

The analysis of secondary outcomes focused on two key variables: the pain during movement (NRS) and the EQ-5D-5L index. These measures were prioritized due to their central role in the study's context—NRS during movement emerged as particularly significant in the primary analysis, and the EQ-5D-5L index provides critical insights into patients' quality of life. Both variables are described in greater detail, reflecting their relevance to the intervention's impact.

While NRS during movement was integrated into the adjusted mixed-effects model, the remaining secondary outcomes were evaluated using both descriptive analyses and significance testing to assess differences between groups. These outcomes include the EQ-5D-5L index, the SF-12 health survey (comprising physical component summary (KSK) and mental component summary (PSK) scales), the PDI, the HADS-D, and the FFbH-R. For these outcomes, box-and-whisker plots visualize distributions, and summary statistics, including *p* values for the significance of differences in mean changes between groups from EVA 1 to EVA 3, are provided to compare data across groups and time points (Table 13). The following sections present the results for each secondary outcome in detail.

**Figure 3:**
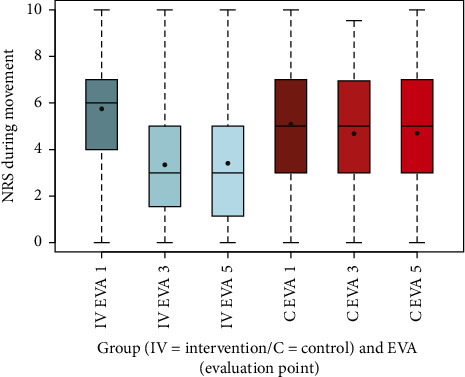
Comparison of NRS during movement intervention (IV) group vs. control (C) group.

The analysis of the NRS during movement, a key secondary outcome, is summarized in box-and-whisker plots (Figure 3) and summary statistics (Table 13). These results compare the intervention (IV) and control (C) groups across the evaluation time points EVA 1, EVA 3, and EVA 5.

At baseline (EVA 1), the intervention group showed higher mean scores (mean: 5.75, SD 2.27) compared to the control group (mean: 5.09, SD 2.74). By EVA 3, a substantial reduction was observed in the intervention group (mean: 3.42, SD 2.42), while the control group exhibited a smaller decrease (mean: 4.66, SD 2.46). This trend persisted at EVA 5, with the intervention group maintaining lower scores (mean: 3.44, SD 2.59), compared to the control group (mean: 4.66, SD 2.40). The difference in mean changes between groups from EVA 1 to EVA 3 was highly significant (*p* < 0.001), underscoring the intervention's effectiveness in reducing dynamic pain.

**Figure 4:**
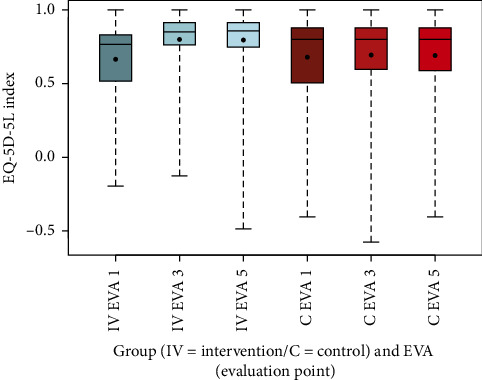
Comparison of EQ-5D-5L index intervention (IV) group vs. control (C) group.

**Figure 5:**
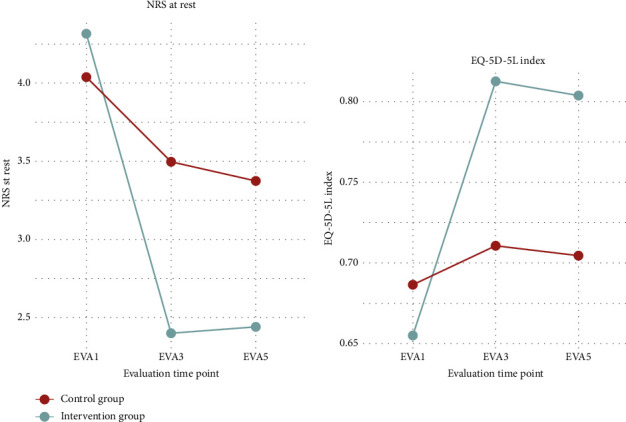
Comparison NRS at rest vs. EQ-5D-5L index, intervention (IV), and control (C) group.

The evaluation of the EQ-5D-5L index, representing health-related quality of life, is presented through box-and-whisker plots (Figure 4), summary statistics (Table 13), and a comparative graphical representation alongside NRS scores at rest over time (Figure 5).

The summary statistics (Table 13) indicate consistent improvements in the intervention group, with the mean EQ-5D-5L index increasing from 0.67 (SD 0.24) at EVA 1 to 0.79 (SD 0.19) at EVA 3, and remaining stable at 0.79 (SD 0.22) at EVA 5. In contrast, the control group showed minimal change, with the index moving from 0.68 (SD 0.28) at EVA 1 to 0.69 (SD 0.29) at EVA 3 and 0.69 (SD 0.28) at EVA 5. The difference in mean changes between groups from EVA 1 to EVA 3 was highly significant (*p* < 0.001), confirming a significant improvement in quality of life in the intervention group compared to the control group.

Box-and-whisker plots (Figure 4) highlight the broader distribution of EQ-5D-5L scores in the control group compared to the more pronounced improvement in the intervention group across all evaluation points. The comparative graphic (Figure 5) shows how the EQ-5D-5L index and NRS at rest change over time, revealing an inverse relationship: As quality of life improves, pain intensity decreases, particularly in the intervention group.

**Figure 6:**
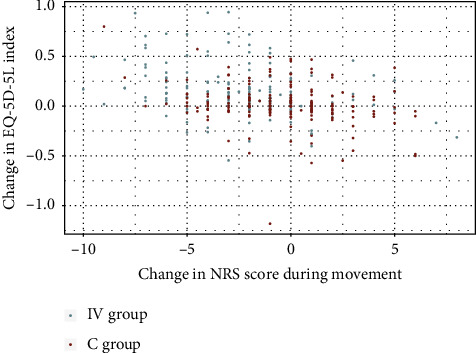
Change in NRS score during movement vs. EQ-5D-5L index, intervention (IV), and control (C) group.

We further investigated the relationship between changes in NRS during movement and EQ-5D-5L index from EVA 1 to EVA 5 for both intervention and control groups. In the intervention group, a moderate, significant correlation was observed between reduced NRS pain scores during movement and increases in the EQ-5D-5L index (*p* < 0.001, *r* = −0.3285), while the control group showed a weaker, yet still significant correlation (*p* < 0.001, *r* = −0.2565) (Figure 6).

**Figure 7:**
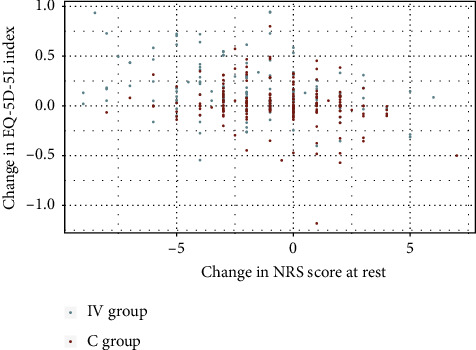
Change in NRS score at rest vs. EQ-5D-5L index, intervention (IV), and control (C) group.

Similarly, both groups exhibited a weak, significant correlation between reduced NRS pain scores at rest and increased EQ-5D-5L index (IV: *p* < 0.001, *r* = −0.2798, C: *p* < 0.05, *r* = −0.1700) (Figure 7).

These correlations suggest that reductions in pain, particularly in the intervention group, are associated with improvements in quality of life; however, this pattern is not unique to the intervention, as similar, though weaker, associations were observed in the control group.

In addition to the analysis of the primary and key secondary outcomes, additional validated instruments were used to assess a broader range of health dimensions in the RütmuS study. These secondary outcomes include the SF-12 Physical Health (KSK) and Mental Health (PSK) Summary Scales, PDI, FFbH-R, and the HADS-D subscales for anxiety and depression. These measures capture complementary aspects of health and functioning, offering a more holistic perspective on patient outcomes.

**Figure 8:**
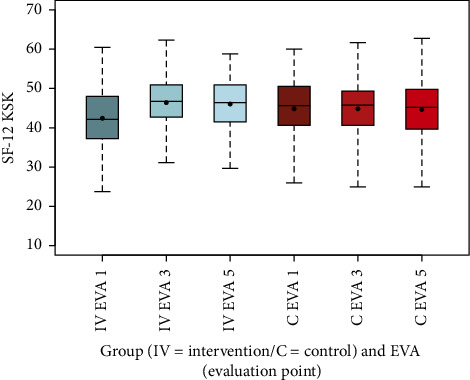
Comparison of SF-12 KSK summary scale intervention (IV) group vs. control (C) group.

**Figure 9:**
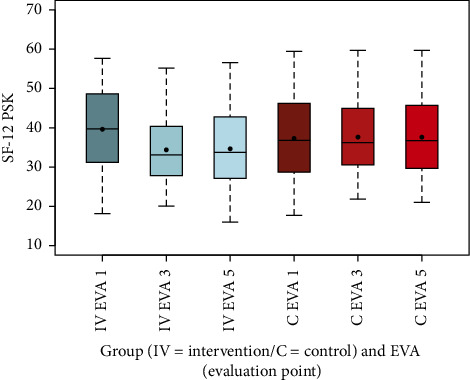
Comparison of SF-12 PSK summary scale intervention (IV) group vs. control (C) group.

The SF-12 KSK summary scale, reflecting physical health, improved in the intervention group from a mean of 42.42 (SD 7.36) at EVA 1 to 46.40 (SD 6.35) at EVA 3 and 46.04 (SD 6.52) at EVA 5, while it remained stable in the control group (EVA 1: 44.82, SD 7.58; EVA 3: 44.82, SD 7.08; EVA 5: 44.64, SD 7.39). This difference in mean changes from EVA 1 to EVA 3 was highly significant (*p* < 0.001). Conversely, the PSK Summary Scale, indicating mental health, decreased in the intervention group from 39.62 (SD 10.41) at EVA 1 to 34.38 (SD 8.80) at EVA 3, recovering slightly to 34.65 (SD 9.62) at EVA 5, while the control group showed a slight increase (EVA 1: 37.29, SD 10.53; EVA 3: 37.63, SD 9.12; EVA 5: 37.61, SD 9.61). This difference was also highly significant (*p* < 0.001), suggesting a potential mental strain in the intervention group (Figures 8 and 9, Table 13).

**Figure 10:**
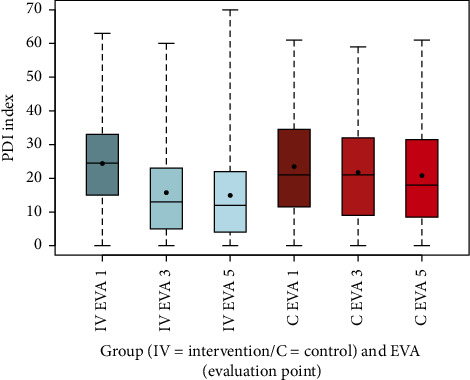
Comparison of PDI index intervention (IV) group vs. control (C) group.

**Figure 11:**
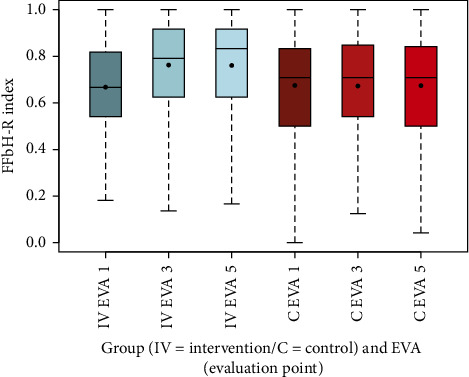
Comparison of FFbH-R index intervention (IV) group vs. control (C) group.

The PDI, measuring perceived disability, showed a substantial reduction in the intervention group from 24.39 (SD 12.86) at EVA 1 to 15.74 (SD 13.35) at EVA 3 and 14.92 (SD 13.94) at EVA 5, compared to a modest decline in the control group (EVA 1: 23.48, SD 15.12; EVA 3: 21.71, SD 14.89; EVA 5: 20.80, SD 14.97). This difference was highly significant (*p* < 0.001) (Figure 10, Table 13). The FFbH-R index, assessing functional ability, improved in the intervention group from 0.67 (SD 0.19) at EVA 1 to 0.76 (SD 0.19) at EVA 3 and 0.76 (SD 0.20) at EVA 5, while remaining stable in the control group (EVA 1: 0.68, SD 0.24; EVA 3: 0.67, SD 0.23; EVA 5: 0.67, SD 0.24), with a highly significant difference (*p* < 0.001) (Figure 11, Table 13).

**Figure 12:**
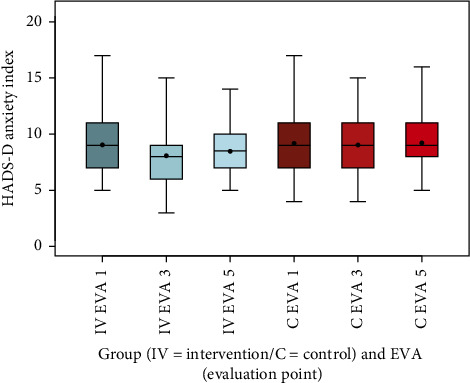
Comparison of HADS-D anxiety index intervention (IV) group vs. control (C) group.

**Figure 13:**
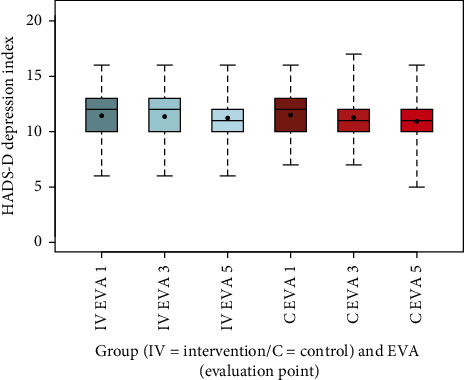
Comparison of HADS-D depression index intervention (IV) group vs. C group.

Mental health outcomes, as assessed by the HADS-D subscales, showed modest changes. The anxiety index decreased in the intervention group from 9 (SD 2.5) at EVA 1 to 8.1 (SD 2.3) at EVA 3, rising slightly to 8.5 (SD 2) at EVA 5, while it remained stable in the control group (EVA 1: 9.2, SD 2.6; EVA 3: 9, SD 2.3; EVA 5: 9.2, SD 2.2). This difference was significant (*p*=0.0164). The depression index showed little variation in both groups (intervention: EVA 1: 11, SD 1.9; EVA 3: 11, SD 1.8; EVA 5: 11, SD 1.7; control: EVA 1: 12, SD 1.7; EVA 3: 11, SD 1.6; EVA 5: 11, SD 1.8), with no significant difference (*p*=0.1093) (Figures 12 and 13, Table 13).

The box-and-whisker plots (Figures 2, 3, 4, 8, 9, 10, 11, 12, 13) visually summarize the outcomes, highlighting group differences and changes over time. Summary statistics in Table 13 provide detailed insights into the patterns observed, showing greater improvements in physical and functional health domains in the intervention group compared to the control group, while mental health outcomes varied, with a notable decline in the PSK scale in the intervention group alongside modest changes in anxiety and depression in both groups.

## 4. Discussion

This observational multicenter RütmuS study demonstrated that the chosen multimodal therapy approach leads to a significant reduction in chronic back pain at rest and during movement, a significant improvement in subjective quality of life, and notable changes in additional health domains, including physical functioning, disability, and mental health outcomes.

As randomization was not feasible due to the regional constraints of the selective contract, it was critical to confirm comparability between the two groups at baseline to ensure valid conclusions. The baseline characteristics outlined in Table 3 reveal that most demographic and clinical parameters did not differ significantly between groups, such as age, gender, BMI, mobility, and self-assessed quality of life. This strengthens the validity of the observed treatment effects, as confounding due to major baseline differences is minimized.

However, some differences were observed. For example, the level of pain during movement was significantly higher in the intervention group at baseline. This difference could lead to bias but also shows the strong effectiveness of the intervention, as the intervention group improved more despite having higher pain levels at the start. The inclusion of baseline pain during movement as a covariate in the adjusted mixed-effects model further mitigates its potential confounding effect.

Additionally, education levels and marital status differed significantly between groups. Although these differences may influence pain perception and coping mechanisms, existing literature provides conflicting evidence on their role in chronic pain outcomes [29, 30]. For this reason, both education level and marital status were included as covariates in the multivariate analysis, aligning with the conceptual framework for selecting potential confounders.

A significant difference was also noted in the distribution of ICD-10 diagnoses, particularly for osteochondrosis of the spine (M42) and other disc damage (M51), both of which were more prevalent in the intervention group. Despite these differences, the primary diagnosis in both groups was back pain (M54), which occurred at similar rates (64.6% vs. 69.7%, *p*=0.282). This supports the generalizability of the findings to patients with chronic back pain. To address these baseline differences and reduce the potential for bias, a mixed-effects model was employed. This approach accounted for random effects at the participant level while incorporating potential confounders, such as education level, marital status, and baseline pain during movement, as fixed effects. By adjusting for these covariates, the model provided a more accurate estimation of the intervention's effects on pain reduction. Furthermore, the model's ability to handle repeated measures within individuals allowed for the evaluation of changes over time while considering individual variability. This ensures that the observed treatment effects are not confounded by baseline differences, thereby enhancing the robustness and reliability of the study's conclusions. Overall, these findings confirm the comparability of the two groups on key variables while acknowledging and addressing significant differences through methodological adjustments. This comprehensive approach strengthens the study's internal validity and ensures that the observed treatment effects can be attributed to the intervention itself. Further studies have demonstrated that MPT can be effective in reducing chronic back pain and improving functional outcomes in patients and a return-to-work [31]. A systematic review and meta-analysis of randomized controlled trials (RCTs) of MPT for chronic back pain found that MPT was superior to monotherapy in reducing pain intensity and improving function [32, 33]. The authors concluded that MPT should be considered the first-line treatment for chronic back pain. Our study results show comparable results. The main hypothesis of the prospective study, that there is a significant difference in the changes in pain intensity (according to NRS at rest) during the evaluation period between the patients of the intervention (MPT therapy) and the control group, could be confirmed. The results, adjusted for a wide range of confounders, provide robust evidence of the intervention's effectiveness in reducing pain at rest. While the analysis primarily focused on pain at rest, the data for pain during movement also indicate favorable outcomes for the intervention group, with a highly significant reduction in mean scores from EVA 1 to EVA 3 compared to the control group (*p* < 0.001). Although baseline pain during movement was higher in the intervention group, subsequent improvements observed over the evaluation period demonstrate the positive impact of the multimodal therapy approach on movement-related pain. Since exercise is considered to have a significant therapeutic effect on chronic low back pain patients [11], it seems to be of great importance for the future treatment of this patient group to offer a multimodal therapy. Lambeek et al. also came to this conclusion in their randomized controlled study [15]. One of the advantages of MPT is that it allows for the use of lower doses of medications, reducing the risk of adverse effects. This is particularly important for opioids, which can be highly addictive and have numerous side effects. By combining different treatments, MPT can provide a more comprehensive and personalized approach to pain management.

Nonpharmacological interventions can help improve strength, flexibility, and mobility, reducing the risk of further injury and improving overall physical function. A Cochrane systematic review by Kamper et al. provides an updated assessment of the effectiveness of multidisciplinary biopsychosocial rehabilitation for chronic low back pain [33]. The review found moderate evidence that multidisciplinary rehabilitation improves pain and function in the short term and may have long-term benefits. It should be noted that there are many different rehabilitative approaches for the treatment of chronic back pain. The investigated approach in the RütmuS study was a moderately elaborate ambulant treatment method. Nevertheless, the intervention group showed a significant reduction in pain for up to 12 months.

The secondary endpoints of this prospective study were designed to complement the primary outcomes by capturing broader dimensions of patient health and well-being. Among these, the EQ-5D-5L was analyzed as a key measure of self-reported quality of life. Findings from the RütmuS study showed that the intervention group experienced greater improvement in quality of life compared to the control group, with a highly significant difference in EQ-5D-5L scores (*p* < 0.001), suggesting that the multimodal therapy approach may contribute to broader health-related quality-of-life dimensions alongside its pain-reducing effects. Chronic pain can have a significant impact on a person's quality of life [34, 35]. It can also cause emotional and psychological distress, including anxiety, depression, and sleep disturbances. Chronic pain can interfere with a person's ability to work, socialize, and enjoy leisure activities, which can further impact their quality of life. In our study, we explored whether reductions in pain correlated with the improvement in quality of life. It could be shown that in both the intervention and the control group, the reduction in the NRS score during movement and at rest correlated significantly with an increase in the EQ-5D-5L index. The fact that the quality of life also correlates with pain in the control group shows how closely these two parameters are linked. The relationship between chronic pain and quality of life is complex, as it can vary depending on a person's individual experiences and circumstances. In general, however, chronic pain is associated with lower levels of physical and emotional well-being and can lead to social isolation and decreased productivity [36].

The results of the RütmuS study demonstrate that the SF-12 score for the physical component (KSK) in the intervention group increased from baseline to EVA 5, with a highly significant improvement compared to the control group, which remained stable (*p* < 0.001). However, the mental component (PSK) decreased overall in the intervention group from EVA 1 to EVA5, with a highly significant difference compared to the control group, which showed a slight increase (*p* < 0.001), indicating potential psychological strain during the intervention period. This finding contrasts with the HADS-D results, where the anxiety index in the intervention group improved from baseline to EVA 5, with a significant difference compared to the control group (*p*=0.0164), while the depression index remained stable in both groups across the same period, with no significant difference (*p*=0.1093). Interestingly, the anxiety improvements were not consistent, showing a temporary decrease at EVA 3 before a slight increase by EVA 5, reflecting opposing trends at midpoints in the study. The complex relationship between chronic pain, psychological distress, and functional impairment is well-documented in the literature. A review by Lee et al. showed that psychological distress and fear influence the relationship between pain and disability [37]. In a longitudinal study of orthopedic outpatients by Kroenke et al., it was shown that pain intensity can influence depression, but that the intensity of depression also influences pain [38]. This interplay underscores the challenges in interpreting the observed trends in psychological outcomes.

Furthermore, the results indicate that pain-related disabilities in daily life, as measured by the PDI questionnaire, and functional limitations in daily activities, assessed by the FFbH-R, both improved in the intervention group over the course of the study, with highly significant differences compared to the control group for both measures (*p* < 0.001). In contrast, the control group showed improvements in the PDI, though less pronounced, while FFbH-R scores remained largely unchanged, with the difference in functional ability being highly significant between groups (*p* < 0.001). These findings suggest that the multimodal intervention may have had a broader impact on both pain management and functional recovery compared to standard outpatient treatment. Effective pain management and coping strategies can improve a person's quality of life and overall well-being. This may include medication, physiotherapy, psychological interventions, lifestyle changes, and support from family and friends [37]. The observed improvements in the intervention group highlight the potential of comprehensive approaches to address the multifaceted challenges of chronic pain. It is essential to work with healthcare professionals to develop an individualized treatment plan that addresses both the physical and emotional aspects of chronic pain. Additionally, the economic implications of implementing MPT for patients with chronic back pain should not be underestimated. Discussions often focus narrowly on the direct costs of therapy, which may initially appear higher for the MPT group when evaluated at this simplistic level.

However, several studies have compared indirect costs in addition to direct costs. MPT may also have economic benefits, as it can reduce healthcare costs associated with chronic back pain. A study conducted in the United States found that a multidisciplinary spine care was associated with a 20% reduction in healthcare costs compared to monotherapy [39].

The findings presented in this analysis are subject to certain limitations. Patient allocation control and intervention groups were determined by regional separation within the study design. While this approach minimized potential biases arising from physicians' recruitment behavior, it precluded achieving a cohort that is representative of the broader German population. Furthermore, for ethical reasons, the allocation of patients to the control group who would have had a regional entitlement to multimodal therapy would not have been acceptable. Given these constraints, incorporating randomization into the study protocol was not a practical option. Another limitation lies in the baseline data, which do not provide information on the duration of the patients' back pain diagnoses or psychological stress prior to enrollment. The literature has shown that earlier treatment of fear and psychological stress can achieve better therapeutic outcomes [40]. It is important to note the absence of data regarding the outpatient treatment received by the control group. The extent to which physiotherapy was prescribed by the attending physician or whether the patients made use of it as a self-funded service remains unclear. Additionally, there is no information on the level of participation in other private sporting activities among patients in either group. These unrecorded variables introduce potential biases that could influence the observed effects beyond the impact of the primary therapy. In conclusion, MPT is an effective and well-tolerated approach to pain management in chronic back pain patients. By combining different treatment modalities, MPT can provide more comprehensive pain relief and improve functional outcomes and quality of life. Healthcare providers should consider MPT as the first-line treatment for chronic back pain and tailor interventions to individual patient needs and preferences. To be able to implement these multimodal therapy approaches in standard care on the basis of evidence, further results are required in terms of medical–economic cost-effectiveness.

## Figures and Tables

**Table 1:** Conservative treatment pathway of the integrated care contract chronic back pain.

Integrated care contract	Physicians	Physiotherapist
Phase I (during first week)	Diagnostic	

Phase II (first to second month)	Control examination (patient-guiding physician) Pain therapy (pain physician)	Complex outpatient therapy (COT) (20 therapeutic units)

Phase III (third to sixth month)	Control examination (patient-guiding physician) Pain therapy (pain physician) Final examination (patient-guiding physician)	Individual physiotherapeutic rehabilitation exercise (24 therapeutic units)

Phase IV (after 12 months)	Follow-up examination (patient-guiding physician)	

**Table 2:** Comparison of the treatment components intervention and control group.

Treatment component	Intervention group	Control group (standard of care)
Physician care	Patient-guiding physician, pain physician, interdisciplinary team	General practitioner/orthopedist
Diagnostics and assessment	Pain anamnesis and psychosocial analysis	Medical history, clinical examination, diagnostic imaging
Physiotherapy	Structured, multimodal therapy with a fixed schedule	Individual prescriptions as required
Education and training	Standardized manual, group sessions	No structured training
Psychosocial consideration	Assessment and group therapy for anxiety, social stress, and depression	No systematic recording
Interdisciplinary cooperation	Weekly case conferences	No coordinated interdisciplinary discussion

**Table 3:** Demographic data and clinical characteristics.

	Total (*n* = 477)	Intervention (IV) (*n* = 243)	Control (C) (*n* = 234)	95% CI	*p*	Missings
Demographic data						
Age (years)	54.44 (±14.48)	53.53 (±14.18)	55.39 (±14.50)	[−4.47, 0.74]	0.257	
Gender (*n* (%) of f./*n* (%) of m.)	333 (69.8)/144 (30.2)	174 (71.6)/69 (28.4)	159 (67.9)/75 (32.1)	[−0.05, 12.31]/[−12.31, 0.05]	0.441/0.441	
BMI (body mass index) (kg)	27.14 (±5.99)	26.85 (±5.52)	27.44 (±6.43)	[−1.67, 0.49]	0.282	
Education (*n* (%))						1 (0.23%)
Postsecondary	75 (15.7)	52 (21.4)	23 (9.8)	[4.74, 18.40]	< 0.05	
Secondary	398 (83.4)	190 (78.2)	208 (88.9)	[−17.69, −3.71]	< 0.05	
Primary	3 (0.6)	1 (0.4)	2 (0.9)	[−2.29, 1.40]	0.974	
Marital status (*n* (%))						
Married or cohabiting	281 (58.9)	134 (55.1)	147 (62.8)	[−16.70, 1.54]	0.107	
Single	94 (19.7)	61 (25.1)	33 (14.1)	[3.54, 18.46]	< 0.05	
Widowed	40 (8.4)	15 (6.2)	25 (10.7)	[−9.91, 0.89]	0.107	
Divorced or separated	62 (13.0)	33 (13.6)	29 (12.4)	[−5.26, 7.64]	0.803	
Clinical characteristics						
Pain at rest at *t*_0_ (NRS)	4.21 (±2.49)	4.37 (±2.32)	4.04 (±2.66)	[−0.12, 0.78]	0.152	
Pain during movement at *t*_0_ (NRS)	5.42 (±2.54)	5.75 (±2.27)	5.09 (±2.75)	[0.20, 1.11]	< 0.05	
FTF (fingertip-to-floor) distance *t*_0_ (in cm)	13.74 (±13.85)	13.53 (±13.05)	13.96 (±14.68)	[−2.94, 2.10]	0.742	5 (1.15%)
Sport and phys. activity (min/week)	129.64 (±161.23)	126.11 (±143.33)	122.32 (±178.24)	[−36.55, 22.12]	0.629	5 (1.15%)
EQ-5D-5L at *t*_0_ (index)	0.673 (±0.261)	0.666 (±0.236)	0.680 (±0.284)	[−0.062, 0.033]	0.551	
ICD—10 (*n* (%))						
M42 (osteochondrosis of the spine)	105 (22.0)	81 (33.3)	24 (10.3)	[15.57, 30.58]	< 0.05	
M50 (cervical disc damage)	48 (10.1)	25 (10.3)	23 (9.8)	[−5.36, 6.28]	0.989	
M51 (other disc damage)	115 (24.1)	87 (35.8)	28 (12.0)	[16.10, 31.58]	< 0.05	
M53 (other diseases of the spine and back, not elsewhere classified)	82 (17.2)	42 (17.3)	40 (17.1)	[−6.77, 7.15]	1.000	
M54 (back pain)	320 (67.1)	157 (64.6)	163 (69.7)	[−13.89, 3.79]	0.282	

*Note:* Demographic data and clinical characteristics; categorization of education: “Postsecondary” includes participants with a university degree (“Hochschulstudium”), “Secondary” represents those with secondary education qualifications such as high school diploma (“Abitur”), advanced technical college entrance qualification (“Fachhochschulreife”), vocational training, lower secondary school certificate (“Hauptschulabschluss”), or intermediate secondary school certificate (“mittlerer Schulabschluss”). “Primary” refers to participants with no formal education (“kein Schulabschluss”). Interpretation of confidence intervals: For metric variables, the 95% confidence intervals indicate the range in which the true difference in arithmetic means between the intervention and control groups is likely to fall. For proportions (nominal variables) presented as percentages, the 95% confidence intervals represent the range in which the true difference in proportions between the groups is expected to lie.

**Table 4:** Evaluation dates and content.

EVA 1 baseline	EVA 2 after 3 months (phone check-in)	EVA 3 after 6 months by post	EVA 4 after 9 months (phone check-in)	EVA 5 after 12 months by post
EQ-5D-5L	EQ-5D-5L	EQ-5D-5L	EQ-5D-5L	EQ-5D-5L
Sports and physical activity (min./week)		Sports and physical activity (min./week)		Sports and physical activity (min./week)
NRS at rest and during movement	NRS at rest and during movement	NRS at rest and during movement	NRS at rest and during movement	NRS at rest and during movement
Fingertip-to-floor		Fingertip-to-floor		Fingertip-to-floor
Birth date				
Gender				
Height and weight				
Marital status				
Education				
ICD code back pain				
SF-12		SF-12		SF- 12
PDI		PDI		PDI
FFbH-R		FFbH-R		FFbH-R
HADS-D		HADS-D		HADS-D

**Table 5:** Fixed effects from the mixed-effects model without confounders.

Effect	Estimate	Std. error	df	*t* value	*p* value
Intercept	4.370	0.150	1035	29.203	< 0.001^∗∗∗^
Group (control)	−0.332	0.214	1035	−1.553	0.121
Time (EIII)	−1.928	0.159	950	−12.114	< 0.001^∗∗∗^
Time (EV)	−1.865	0.159	950	−11.715	< 0.001^∗∗∗^
Group: Time (EIII)	1.353	0.227	950	5.955	< 0.001^∗∗∗^
Group: Time (EV)	1.294	0.227	950	5.695	< 0.001^∗∗∗^

*Note:* Significance levels: ^∗∗∗^*p* < 0.001.

**Table 6:** Post hoc pairwise comparisons for group differences at each time point (Bonferroni adjustment) for mixed-effects model without confounders.

Time	Contrast	Estimate	Std. error	df	*t* value	*p* value
EI	Intervention–control	0.332	0.214	1035	1.553	0.121
EIII	Intervention–control	−1.022	0.214	1035	−4.781	< 0.001^∗∗∗^
EV	Intervention–control	−0.962	0.214	1035	−4.505	< 0.001^∗∗∗^

*Note:* Significance levels: ^∗∗∗^*p* < 0.001.

**Table 7:** Estimated marginal means (95% CI) for mixed-effects model without confounders.

Time	Group	Mean	SE	df	Lower 95% CI	Upper 95% CI
EI	Intervention	4.37	0.150	1035	4.08	4.66
EI	Control	4.04	0.153	1035	3.74	4.34
EIII	Intervention	2.44	0.150	1035	2.15	2.74
EIII	Control	3.46	0.153	1035	3.16	3.76
EV	Intervention	2.51	0.150	1035	2.21	2.80
EV	Control	3.47	0.153	1035	3.17	3.77

**Table 8:** Fixed effects from the mixed–effects model with confounders.

Effect	Estimate	Std. error	df	*t* value	*p* value	Global *p* value
Intercept	2.597	0.580	502	4.480	< 0.001^∗∗∗^	
Group (control)	0.053	0.195	1081	0.271	0.786	
Time (EIII)	−0.818	0.159	1049	−5.148	< 0.001^∗∗∗^	
Time (EV)	−0.769	0.159	1048	−4.842	< 0.001^∗∗∗^	
Age	−0.010	0.006	465	−1.688	0.092^.^	
Gender (female)	0.246	0.162	457	1.521	0.129	
BMI	−0.018	0.012	458	−1.455	0.146	
NRS movement	0.441	0.023	1401	19.416	< 0.001^∗∗∗^	
Education (overall)						0.396
Education (vocational training)	0.260	0.270	456	0.962	0.337	
Education (advanced technical college entrance qualification)	−0.167	0.306	459	−0.547	0.585	
Education (lower secondary school certificate)	0.186	0.311	456	0.598	0.550	
Education (university degree)	−0.069	0.289	459	−0.238	0.812	
Education (no degree)	1.700	0.939	456	1.810	0.071^.^	
Education (no response)	−0.122	1.575	454	−0.077	0.938	
Education (middle school degree)	0.032	0.280	460	0.113	0.910	
Marital status (overall)						0.864
Marital status (separated)	0.391	0.562	456	0.695	0.487	
Marital status (cohabiting)	−0.133	0.399	455	−0.333	0.739	
Marital status (single)	−0.054	0.285	457	−0.189	0.850	
Marital status (married)	−0.191	0.243	459	−0.786	0.432	
Marital status (widowed)	−0.148	0.337	455	−0.438	0.662	
ICD code M42 (yes)	0.260	0.164	1374	1.583	0.114	
ICD code M50 (yes)	0.040	0.176	1382	0.229	0.819	
ICD code M51 (yes)	0.060	0.130	1388	0.460	0.646	
ICD code M53 (yes)	−0.031	0.134	1355	−0.228	0.820	
ICD code M54 (yes)	0.035	0.107	1391	0.330	0.742	
Group × time (EIII)	0.440	0.214	982	2.059	0.040^∗^	
Group × time (EV)	0.411	0.213	980	1.925	0.055^.^	

*Note:* Global *p* values are provided for categorical predictors with more than two levels. Significance levels: ^∗∗∗^*p* < 0.001, ^∗^*p* < 0.05, and ^.^*p* < 0.1. For categorical variables, one level serves as the reference category, and the effects of other levels are shown relative to this reference. The reference categories are not explicitly displayed in the table but are defined based on the coding of the model.

**Table 9:** Random effects from the mixed–effects model without confounders.

Group	Variance	Std. dev.
Participant (intercept)	2.364	1.537
Residual	3.079	1.755

**Table 10:** Random effects from the mixed–effects model with confounders.

Group	Variance	Std. dev.
Participant (intercept)	1.542	1.242
Residual	2.528	1.590

**Table 11:** Post hoc pairwise comparisons for group differences at each time point (Bonferroni adjustment) for mixed-effects model with confounders.

Time	Contrast	Estimate	Std. error	df	*t* value	*p* value
EI	Intervention–control	−0.053	0.195	1084	−0.271	0.786
EIII	Intervention–control	−0.492	0.191	1061	−2.576	0.010^∗^
EV	Intervention–control	−0.463	0.191	1060	−2.429	0.015^∗^

*Note:* Significance levels: ^∗^*p* < 0.05.

**Table 12:** Estimated marginal means (95% CI) for mixed-effects model with confounders.

Time	Group	Mean	SE	df	Lower 95% CI	Upper 95% CI
EI	Intervention	4.06	0.299	631	3.47	4.65
EI	Control	4.11	0.300	684	3.52	4.70
EIII	Intervention	3.24	0.303	656	2.65	3.84
EIII	Control	3.73	0.297	670	3.15	4.32
EV	Intervention	3.29	0.302	650	2.70	3.88
EV	Control	3.75	0.298	677	3.17	4.34

**Table 13:** Summary statistics of primary and secondary outcomes.

Outcome	Group	Mean			SD			Min	Max	*p* value
		EVA1	EVA3	EVA5	EVA1	EVA3	EVA5			
NRS pain at rest	Intervention	4.37	2.43	2.48	2.32	2.29	2.24	0.00	10.00	< 0.001^∗∗∗^
	Control	4.04	3.39	3.39	2.65	2.20	2.17	0.00	10.00	

NRS pain during movement	Intervention	5.75	3.42	3.44	2.27	2.42	2.59	0.00	10.00	< 0.001^∗∗∗^
	Control	5.09	4.66	4.66	2.74	2.46	2.40	0.00	10.00	

EQ‐5D‐5L index	Intervention	0.67	0.79	0.79	0.24	0.19	0.22	−0.49	1.00	< 0.001^∗∗∗^
	Control	0.68	0.69	0.69	0.28	0.29	0.28	−0.58	1.00	

SF‐12 KSK summary scale	Intervention	42.42	46.40	46.04	7.36	6.35	6.52	23.76	62.32	< 0.001^∗∗∗^
	Control	44.82	44.82	44.64	7.58	7.08	7.39	24.99	62.72	

SF‐12 PSK summary scale	Intervention	39.62	34.38	34.65	10.41	8.80	9.62	16.01	57.67	< 0.001^∗∗∗^
	Control	37.29	37.63	37.61	10.53	9.12	9.61	17.70	59.69	

PDI index	Intervention	24.39	15.74	14.92	12.86	13.35	13.94	0.00	70.00	< 0.001^∗∗∗^
	Control	23.48	21.71	20.80	15.12	14.89	14.97	0.00	61.00	

FFbH‐R index	Intervention	0.67	0.76	0.76	0.19	0.19	0.20	0.14	1.00	< 0.001^∗∗∗^
	Control	0.68	0.67	0.67	0.24	0.23	0.24	0.00	1.00	

HADS‐D anxiety index	Intervention	9	8.1	8.5	2.5	2.3	2	3	17	0.0164^∗^
	Control	9.2	9	9.2	2.6	2.3	2.2	4	17	

HADS‐D depression index	Intervention	11	11	11	1.9	1.8	1.7	6	16	0.1093
	Control	12	11	11	1.7	1.6	1.8	5	17	

*Note:* Summary statistics (mean, SD, min. and max.) for all evaluated indices over the evaluation time points (EVA1, EVA3 and EVA5) for the intervention and control groups. The values in the last column represent the significance of the difference in the mean changes between the intervention (IV) and control (C) groups from EVA1 to EVA5. Significance levels: ^∗∗∗^*p* < 0.001 and ^∗^*p* < 0.05.

## Data Availability

The data that support the findings of this study are available from the corresponding author upon reasonable request.
